# Rich production media as a platform for CHO cell line development

**DOI:** 10.1186/s13568-020-01025-3

**Published:** 2020-05-15

**Authors:** Yong Jae Kim, Sang Kyul Han, Seongtae Yoon, Chan Wha Kim

**Affiliations:** 1 Department of Biotechnology, College of Life Sciences and Biotechnology, Korea University, 145, Anam-ro, Seongbuk-gu, Seoul, 02841 Republic of Korea; 2 GC Pharma R &D Center, GC Pharma, 93, Ihyeon-ro 30 beon-gil, Giheung-gu, Yongin-si, Gyeonggi-do 16924 Republic of Korea

**Keywords:** Cell line development, Chemically defined media, Single cell isolation, Semi-solid media, Chinese hamster ovary cell

## Abstract

Recent cell culture media for mammalian cells can be abundantly formulated with nutrients supporting production, but such media can be limited to use in host cell culture, transfection, cell cloning, and cell growth under the low cell density conditions. In many cases, appropriate platform media are used for cell line development, and then replaced with rich media for production. In this study, we demonstrate rich chemically defined media for Chinese hamster ovary (CHO) cells that are suitable as basal media both for cell line development and for final production of culture process. Set up for transfection, semi-solid media optimization, mini-pool screening, and single cell cloning media development were performed, and final clones were obtained with higher productivity in fed-batch culture mode using rich formulated media comparing with lean formulated media. Developed methods may remove the requirements for cell adaptation to production media after cell line development, and relieve the clonality issues associated with changing the culture media. Furthermore, established methods have advantages over traditional approaches, including saving resources and decreasing the time and the effort required to optimize the production process.

## Introduction

The cell culture media are necessary to supply sufficient nutrients for the cell growth for mammalian cells such as Chinese hamster ovary (CHO) cells, and applying appropriate media is important in developing cell line and cell culture process with high productivity and quality. Media have been developed during the past decades, which have led to changes in process aspects of cell line development (CLD). Generally, the media are classified as the types of serum media (SM), serum-free media (SFM), protein-free media (PFM), and chemically defined media (CDM), depending on the components of the media. Traditional cell line development was performed in serum supplemented classical media, which gave problems in the production process development such as low productivities (below 100 mg/L) due to the poor media, the issues in developing adherent cell culture process, and the issues due to the use of serum (Glassy et al. 1988; Keen and Rapson 1995; Gstraunthaler et al. 2013).

**Fig. 1:**
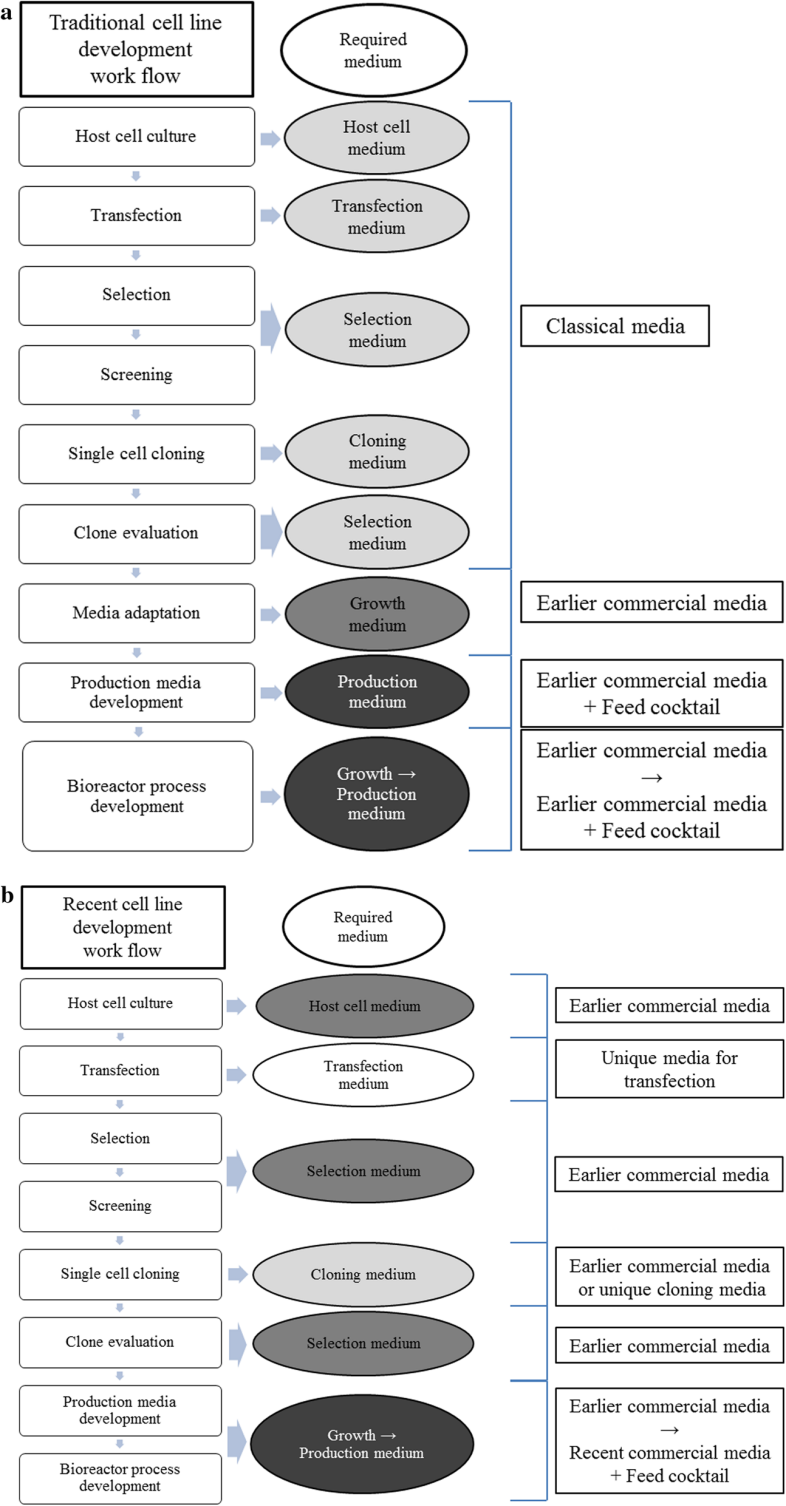

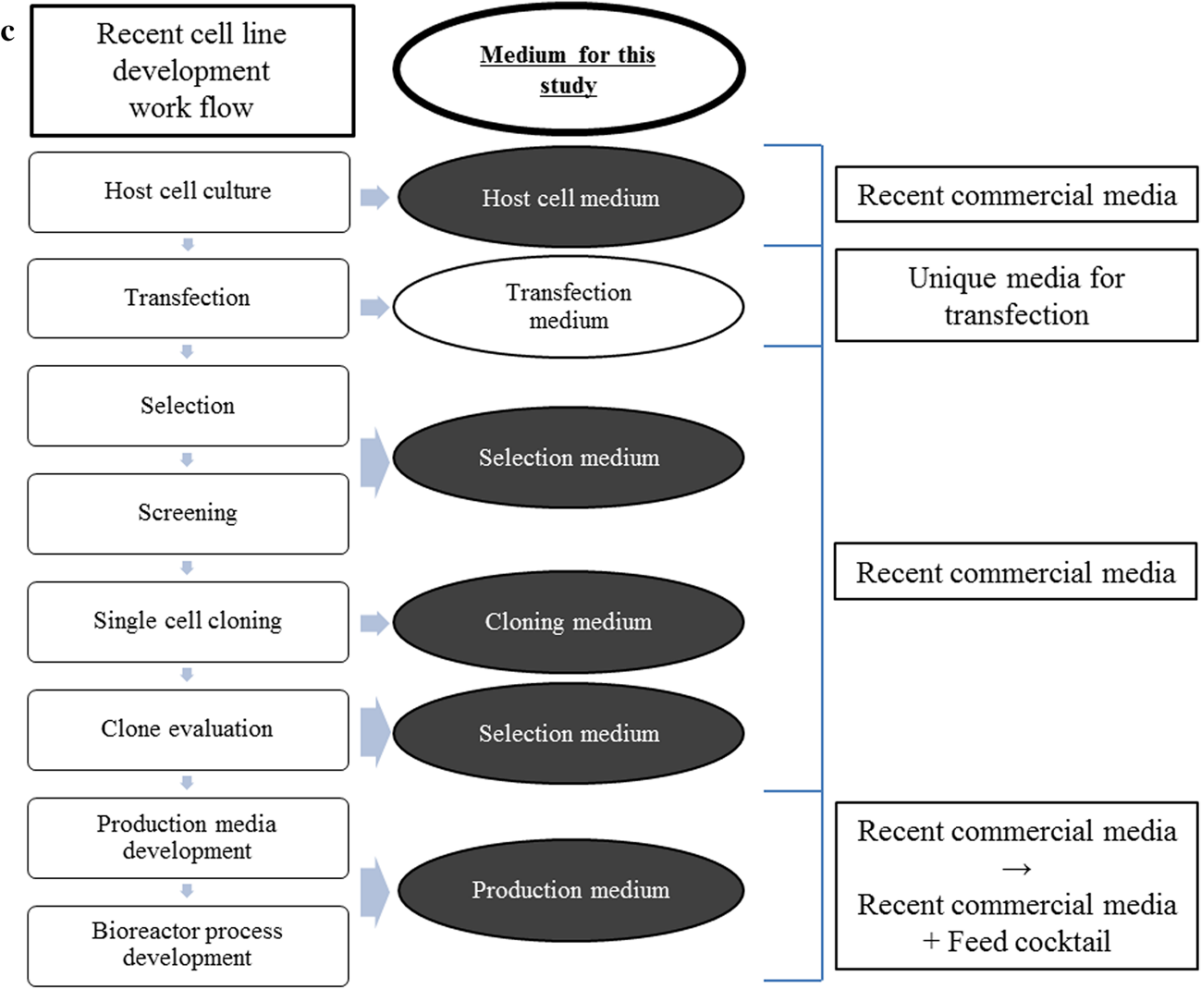
Typical cell line development (CLD) workflow change following media development (**a**) traditional CLD using classical media (with serum) during cell line screening and then adapting to (serum-free) commercial media, ((**b**) recent CLD changes by adapting host cells to commercial media and using throughout the CLD process, however transfection and cloning requires a unique media, while production will mostly require a rich production media (recent commercial media) to achieve higher productivities, **c** our intended CLD method in this study which uses a rich production media (recent commercial media) as platform media throughout the study minimizing use of different media from the platform media

After SFM was available, the cell line was adapted to SFM to boost up the productivity, apply to suspension cell culture process and relieve the issues using serum (Fig. 1a). Current CLD commonly simplifies the process by employing SFM-adapted host cells and developing the cells in serum-free conditions in order to reduce the time of adapting cells in SFM at the later stage (Fig. 1b). However, technically, this requires optimization of the host cell media, transfection media, selection media, and the cloning media used in each step of CLD. Therefore, setting up a new CLD platform with SFM consumes the significant resources to optimize the media in each step and may use the different media from the basal platform media in a particular step.

In present, newly developed cell culture processes tend to use recent CHO media in rich nutrients for the final production in bioreactor to increase the likelihood of high productivity. The media compositions of commercial formulation are not available in the public and are limited to the individuals related in media development field. However, the good indicator of the media richness may be amino acids, which are commonly assessed in the cell culture field. Herein, we assessed the amino acid contents in the media described in the literature or analyzed internally, and identified an increasing trend of amino acid contents in the media over time that generally appears to be correlated with increased productivity (Table 1) (Huang et al. 2010; Takagi et al. 2017; Reinhart et al. 2015; Carrillo-Cocom et al. 2015; Pan et al. 2017). Therefore, we made a new classification for the media in this study, which distinguishes the classical media (media which are insufficient to grow cells without supplementation), earlier commercial media (with relatively lean nutrients), and the recent commercial media (with relatively rich nutrients).

**Table 1:** Media features and amino acid content ranges

Media classification in this study	Classical media (MEM-a, DMEM, F12, RPMI 1630, etc.)	Earlier commercial CHO media	Recent commercial CHO media
Media characteristics	Basal media used with serum addition or elementary level serum-free media adding some supplements	Serum-free, protein-free media, lean CDM	Mostly CDM
Years started to be employed	1960s–1980s	1990s–2000s	2000s–2010s
Typical productivity^a^	Up to 0.1 g/L	Up to 1 g/L	1–10 g/L
Amino acid content range^b^ (excluding glutamine)	0.5–1 g/L	1–3 g/L	3–12 g/L

^a^References: Huang et al. (2010) and Takagi et al. (2017)

^b^Formulation of classical media is publically available. Amino acid content is the expected range based on internal amino acid analysis and/or references (Reinhart et al. 2015; Carrillo-Cocom et al. 2015; Pan et al. 2017). Amino acids are detected (and reported in publications) as molar mass, so the amino acid concentration is calculated with molecular weight

In most cases, CLD is performed in leaner platform media (earlier commercial media) and then changing the production media to the new richer media (recent commercial media). However, recent regulatory perspectives of US Food and Drug Administration (FDA) have emphasized the importance of the cell line clonality (Welch and Arden 2019). CHO cells are known to be genomically unstable and heterogeneous which leads to frequent genotypic variation and phenotypic instability, so regulatory authority concerns that this may not bring process reproducibility or consistency (Bailey et al. 2012; Li et al. 2016; Baik and Lee 2017). For this reason, the production cell line is required to be isolated from a single cell, and the cloning process should be performed in CLD for this purpose. Additionally, FDA points out recently that cloning should be performed after an adaptation of cells to serum-free or suspension culture conditions, and an additional cloning is required when post-cloned cells are adapted to new conditions (Welch and Arden 2019). Also, the most recent report highly concerns the genetic diversity, and the spontaneous mutations in CHO cells can rapidly occur in a very short period (risk of diversity even in a cell division) (Wurm and Wurm 2017).

To mitigate this risk, we attempted to establish a new platform using recent commercial CHO media from the beginning of CLD and to make the changes of media least (Fig. 1c). We generally switched to richer media for the production after a cell line was established with lean media in the past. However, after CHO host cells were pre-adapted to each production media candidates, we continued CLD using each production media candidates throughout its workflow, and evaluated the screened clones in this study.

## Materials and methods

### Cells and culture media

CHO-DG44 cells obtained from Dr. Chasin (Columbia University, New York, USA) were serum-free-adapted in CDM4CHO (GE Healthcare, Björkgatan, Uppsala, Sweden) and used as host cells for CLD. CDM4CHO and two other chemically defined media, HyCell CHO (GE Healthcare, Björkgatan, Uppsala, Sweden) and ActiPro (GE Healthcare, Björkgatan, Uppsala, Sweden), were each tested as basal media in CLD with a cell line producing IgG. CDM4CHO will be stated as CDM1, HyCell CHO as CDM2, and ActiPro as CDM3 for the convenience in the text.

Before the study, host cells to be used in CDM2 and CDM3 experiments were adapted for ~ 20 subcultures with CDM1-adapted host cells before a use in CLD basal media comparison studies. 100× hypoxanthine and thymidine (HT) supplement (Thermo Fisher Scientific, Waltham, MA, US) was added at 2× in each media to grow the host cells, and 6 mM of l-Glutamine (Thermo Fisher Scientific, Waltham, MA, US) was added as well.

### Transfection and cell culture

In CLD, host cells were sub-cultured for adaptation to the media (CDM1, CDM2, or CDM3) under suspension conditions in shake flasks (Merck KGaA, Darmstadt, Germany) (shaking at 140 rpm, 37 °C, 5% CO_2_, inoculum cell density of 3–5 × 10^5^ cells/S, 2–3 day subculture) until the cells were healthy (viability > 95%). Media were temporarily changed to OptiPRO SFM (Thermo Fisher Scientific, Waltham, MA, US) when transfecting the IgG-expressing plasmid using 1 mg/mL polyethylenimine Mw 40,000 Da (Polyscience, Inc, Warrington, PA, USA). Transfected pool was then transferred back to its original basal media (CDM1, CDM2, or CDM3) and cultured under suspension conditions for 48 h in the presence of HT. After 48 h, cells were either used for mini-pool assessment or ClonePix2 (Molecular Devices, San Jose, CA, USA) experiments. The cells were further cultured without HT and adding 20 nM Methotrexate (MTX) until the full recovery of viability (> 90%) to check the productivity of the transfected pool.

### Clones screening and IgG quantification

The clones with mini-pool assessment or ClonePix2 experiments were screened by evaluating the productivity using an Octet^®^ QKe system (ForteBio, Fremont, CA, USA). Mini-pools were formed by seeding 1000–4000 transfected cells/well in 96-well plates and culturing for 2–3 weeks at 37 °C in a 5% CO_2_ incubator. 20 nM MTX was added to basal media at this step for the amplification of gene of interest. Supernatant from wells forming mini-pools was assessed by using an Octet^®^ QKe system to measure IgG productivity. In the case of ClonePix2 assessment, transfected cells were cultured for 12–16 days at 37 °C in a 5% CO_2_ incubator in semi-solid media prepared by mixing concentrated basal media (2× concentrate for CDM1, 1.5× concentrate for CDM2 and CDM3) with CloneMatrix reagent (Molecular Devices, San Jose, CA, USA). The final preparation containing 20 nM MTX and recombinant CloneDetect reagent (Molecular Devices, San Jose, CA, USA; a fluorescein-labeled antibody with high affinity and specificity for human IgG) was inoculated into 6-well plates with 20,000 transfected cells/mL. Clones were formed in semi-solid media at the same point of exhibiting fluorescence when expressing the target IgG, and the intensely fluorescent clones were picked and transferred to 96-well plates, and supernatants were also assessed by using an Octet^®^ QKe system after 3–7 days of culturing.

Clones expressing the highest IgG productivity in mini-pool or ClonePix2 experiments in 96-well microplates were further cultured in 24-well and 6-well plates. A final productivity check was performed with using 6-well plates after 3 days at 37 °C in 5% CO_2_ incubator starting from the fixed seeding density. Cell density was measured using a CEDEX HiRes Analyzer (Roche, Mannheim, Germany) to calculate cell-specific productivity from the Octet^®^ QKe results.

Finally, candidate cell lines were identified after single cell isolation by limited dilution cloning (LDC) and analysis of single clones using an Octet^®^ QKe in 96-well and 6-well plates. Cell image was analyzed using a Clone Select Imager (Molecular Devices, San Jose, CA, USA) to observe the wells with single isolated cells and growing into colonies.

### Final clone evaluation

Top clones screened from each media were tested in batch culture mode using shake flasks or in fed-batch culture mode using Ambr^®^ 15 microscale bioreactors (Sartorius, Royston, UK). Batch culture mode was performed adding glucose to be maintained above approximately 2 g/L during the culture period. In the case of fed-batch culture mode, cells were fed sequentially four times every 2 days after the first feed-point, day 3, with 4% of Cell boost 7a and 0.4% of 7b (GE Healthcare, Björkgatan, Uppsala, Sweden) following the manufacturer’s recommendation and protocol, and temperature was shifted from 37 to 32 °C after the first feed. Cell-specific productivity (Qp) is calculated based on the growth rate (μ) and the volumetric productivity.

The growth rate (μ) was calculated as follow:

$$ \mu = \frac{lnN2 - lnN1}{t2 - t1} $$

where N is the viable cell density and t is the cultivation time.

The cell-specific productivity was calculated as follow:

$$ Qp = \mu *\frac{P2 - P1}{N2 - N1} $$

where P is the volumetric productivity.

## Results

### Host cell adaptation and transfection

We tested some of the recent commercial CHO media developed by major companies to evaluate the media which work well with our CLD platform (e.g. host cell, vector) (Table 2). CDM2, CDM3, and CD FortiCHO (Thermo Fisher Scientific, Waltham, MA, US) worked well in our monoclonal antibody cell line and recombinant protein cell line which are developed from our CLD platform using CDM1. We attempted to adapt CHO-DG44 cells already adapted to CDM1 to these media, and cells only failed to adapt to CD FortiCHO (data not shown). After transfecting the IgG-containing plasmid using polyethylenimine, productivity after culturing for 48 h was measured, and results between trials were diverse (Table 3). The reasons for this variability are not known, but may be related to the state of the host cell preparation. However, when transfected pools were continually sub-cultured to recover the cell growth, the adapted pool displayed similar batch results, suggesting that the results of productivity are not directly related to the success or failure of transfection all the time. CDM3 yielded lower productivity at 48 h after the transfection in each trial. After the adaptation, CDM2 and CDM3 achieved higher productivity during batch culturing of pools, presumably due to the increased supply of components in these richer media (Table 3).

**Table 2:** Evaluation of media performance based on platform media

Media candidates^b^	Relative productivity for IgG^d^	Relative productivity for recombinant protein^d^	Cell aggregation^d^	Total amino acid content range (excluding glutamine)^a^
CDM1^c^ (CDM4CHO)	Moderate	Moderate	Negligible	2.0–3.0
CDM2 (HyCell CHO)	High	High	Negligible	4.5–5.5
CDM3 (ActiPro)	High	Moderate	Negligible	6.5–7.5
CD FortiCHO	High	High	Usually observed	7.5–9.0
Cellvento CHO-210	Low	High	Frequently observed	3.5–4.5
ExCell Advanced CHO	Moderate	Low	Negligible	7.0–8.0
PowerCHO-2	Moderate	Poor	Usually observed	10.5–12.5

^a^Expected ranges are from internal amino acid analysis and/or references (Reinhart et al. 2015; Carrillo-Cocom et al. 2015; Pan et al. 2017). Amino acids are detected (and reported in publications) as molar mass, so the amino acid concentration is calculated with molecular weight

^b^CDM4CHO (GE Healthcare, Björkgatan, Uppsala, Sweden), HyCell CHO (GE Healthcare, Björkgatan, Uppsala, Sweden), ActiPro (GE Healthcare, Björkgatan, Uppsala, Sweden), CD FortiCHO (Thermo Fisher Scientific, Waltham, MA, US), Cellvento CHO-210 (Merck KGaA, Darmstadt, Germany), ExCell Advanced CHO (SAFC Bioscience, St, Louis, Mo, USA), PowerCHO 2 (Lonza, Verviers, Belgium)

^c^CDM1 is an earlier commercial CHO media. The rest are recent commercial CHO media according to our classification

^d^The cell lines tested here are developed from our CLD platform using CDM1, the results of each media here may be different in other cell lines

**Table 3:** Productivities after transfection

	CDM1	CDM2	CDM3
48 h after transfection	0.4–7.9 mg/L	1.0–9.2 mg/L	0.2–4.3 mg/L
Adapted pool batch (day 3)	8.5–12.6 mg/L	12–13.5 mg/L	5.3–10.2 mg/L
Adapted pool batch (day 7)	24.7–37.5 mg/L	86–88 mg/L	86–102 mg/L
Adapted pool batch (day 11)	Not detected^a^	90–110 mg/L	175–210 mg/L

^a^Not detected at the indicated time point because the CDM1-adapted pool batch culture was terminated on day 7

### Comparison of media using mini-pool cloning experiments

Growing mini-pools in 96-well plates is a general practice in CLD for screening cells before the non-producer cells outgrow the producer cells during a recovery after transfection. Our previous CLD process in CDM1 was efficient for growing mini-pools when inoculating cells from 48 h after transfection at 1000–4000 cells/well, depending on the transfected gene, and 2000 cells/well were optimal for growing mini-pool clones. For these IgG-producing cells, ~ 10% of seeded wells, subsequently yielded mini-pool clones after 2–3 weeks of culture, which is a typical degree of recovery for this process (Table 4). In the case of CDM2, inoculation with 1000, 2000, or 4000 cells/well yielded an extremely high growth ratio regardless of the seeding conditions, with 96% of wells yielding growth across all plates tested (Table 4). By contrast, CDM3 resulted in no growth, even with inoculation at high cell densities (Table 4).

**Table 4:** Summary of mini-pool screening results

	CDM1	CDM2	CDM3
Inoculum conditions	2000 cells/well	1000–4000 cells/well	2000–4000 cells/well
Total wells	960	448	960
Growth wells	100	432	0
Growth ratio (%)	10.4	96.4	0
Picked wells (> 10 mg/L)	32	122	0
Picked/Growth ratio (%)	32	28.2	0

In the case of CDM1, too many seeded cells may initially display a high growth ratio, but the cells eventually exhibit the shorter longevity after some clones suffer from the damage due to nutrient depletion. Meanwhile, CDM2 was much more efficient, not only for generating mini-pools, but also for maintaining clones for a longer period. However, further checks are needed to determine whether these conditions are also capable of growing non-producers, which may not be the case since the Picked/Growth ratio was similar (~ 30%). However, the criterion for only picking wells > 10 mg/L may be problematic because CDM2 is richer media, and the higher volumetric productivity would be expected at lower cell-specific productivity compared with CDM1. Interestingly, the poor growth in CDM3 could indicate that rich media are not useful for growing cells at low cell densities.

### Comparison of media using ClonePix2 cloning experiments

The media application to clone screening was also tested in more up-to-date ClonePix2 technology. A semi-solid preparation was prepared according to the ClonePix2 manual and used to grow and detect the high producer colonies for CLD. Identifying conditions in which colonies can grow well in semi-solid media is critical. Two products were provided by the vendor, and CloneMedia reagent (CloneMatrix and mixture of venders media) was found to work well with various cells and conditions, but technically, the detected high producers could be variable when they return to their original media. However, CloneMatrix reagent can be used by mixing cell culture media in a concentrated form, which requires optimization, but was applied because it can be cultured with its original adapted cell media and can avoid potential issues due to the media change. We tested various media and found that some components in rich media were unable to dissolve when concentrated 2× , while some media failed to yield colonies when inoculated with 200–1000 cells/mL (data not shown). In these cases, we found that formulating some rich media at 1.5× concentration solved this problem and resulted in sufficient colony growth.

**Fig. 2:**
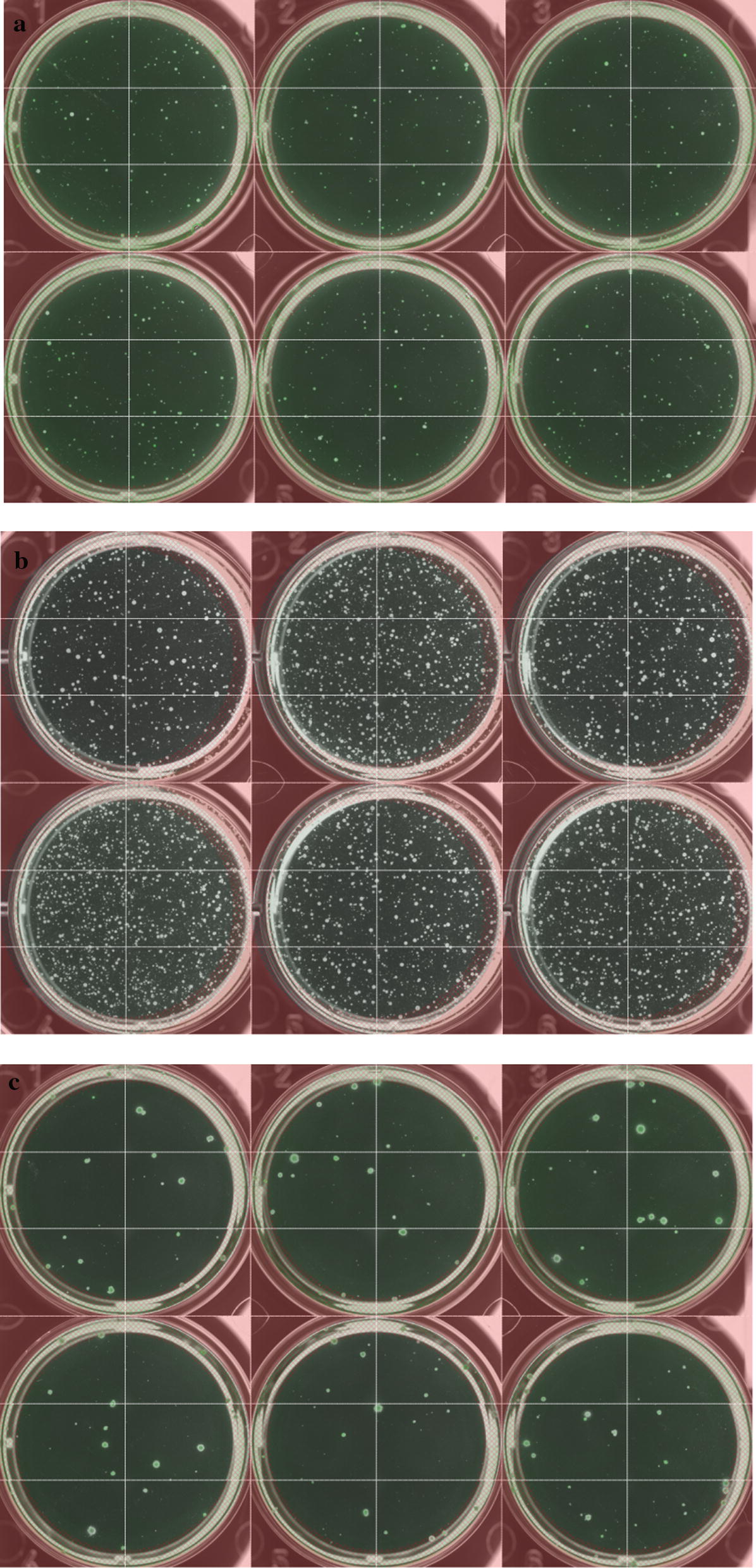
ClonePix2 images comparing colonies from **a** CDM1/CloneMatrix mixtures, **b** CDM2/CloneMatrix mixtures, and **c** CDM3/CloneMatrix mixtures

The CloneMatrix preparation was inoculated with using cells from the 48 h transfected pools generated from CDM1, CDM2 and CDM3 adapted cells (Fig. 2; Table 5). CDM1 yielded the most pickable number of clones achieving productivity above ~ 10 mg/L in 96-well plates. CDM2 yielded numerous total clones, but most of the portions of colonies were too small or located too close to nearby clones (proximity clones), and hence unsuitable for picking. Meanwhile, CDM3 suffered from the poor overall clone growth. CDM3 also suffered from the poor recovery of clones grown in 96-well plates, and few could be successfully scaled up for culturing in 6-well plates. Picked clones from CDM2 showed good growth, and many high-producing clones (> 10 mg/L) identified in 96-well plates were successfully screened and scaled up to 6-well plates. However, since CDM2 is richer than CDM1, the cell-specific productivity analysis in 6-well plates was required to establish whether high-producing clones grown in CDM2 resembled those grown in CDM1.

**Table 5:** Summary of ClonePix2 cloning results

Conditions	CDM1^a^	CDM2	CDM3
Too small (excluded colonies too small)	3822	6483	728
Proximity (excluded colonies too close to each other)	2345	4768	156
Pickable colonies	3863	641	128
Picked colonies (96-well clones)	448	256	128
Screened and picked clones (96-well clones > ~ 10 mg/L)	168	84	NA^b^
6-well clones	120	81	17

^a^CDM1 ClonePix2 experiments using fully recovered pool-screened clones in 96-well plates > ~ 5 mg/L

^b^Not applied because colonies of the recovered clones from 96-well plates were too small

### Single cell isolation and comparison of 6-well data

**Fig. 3:**
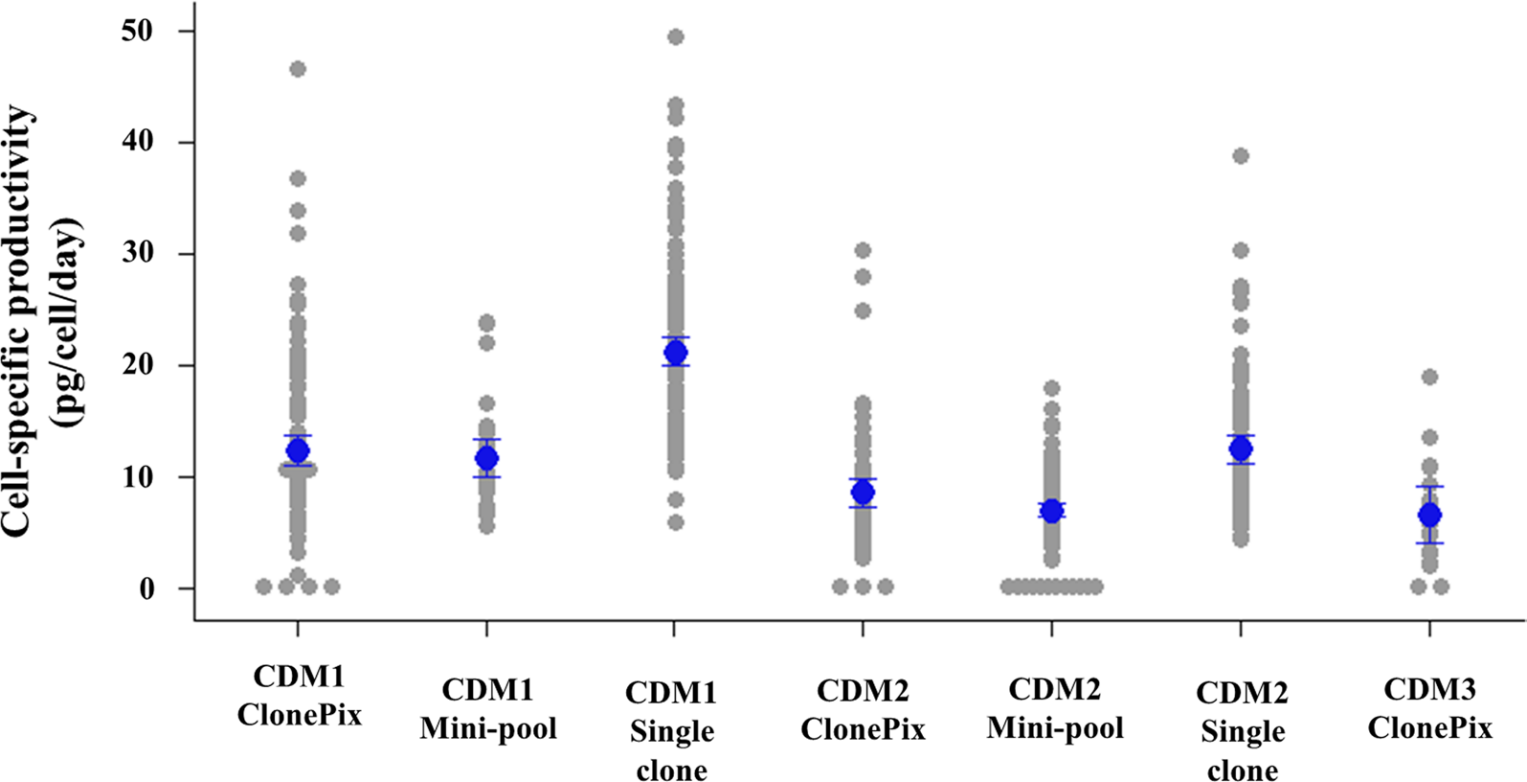
Cell-specific productivity from 6-well plates

All clones screened from using mini-pool and ClonePix2 methods were scaled up to 6-well plates to compare the cell-specific productivity under the uniform culture conditions (Fig. 3). Top five clones from CDM1 and CDM2 were selected for the single cell isolation. CDM3 clones were not selected because the productivity was insufficient.

**Fig. 4:**
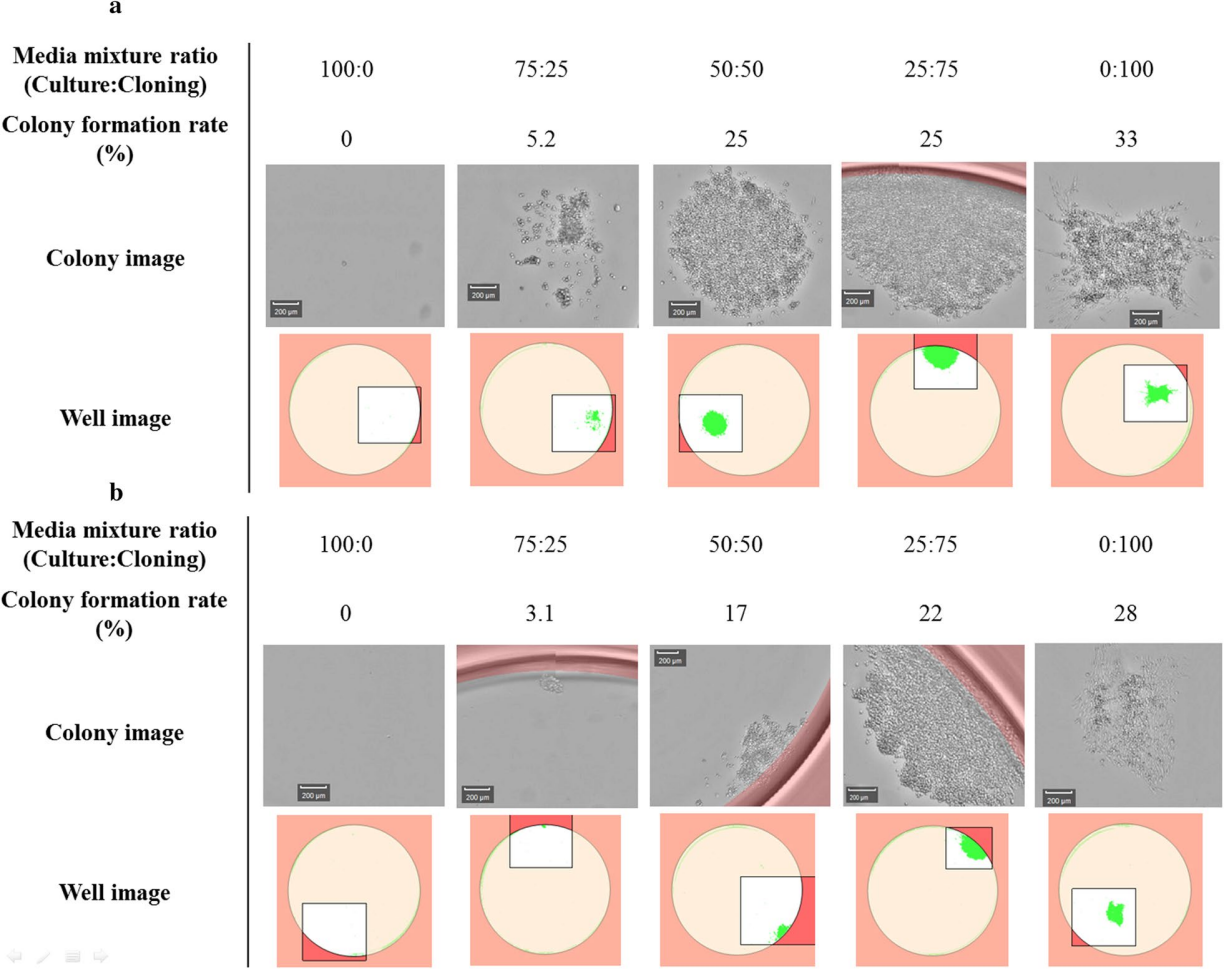
Testing mixtures of commercial cloning media and CDM2 to achieve successful single cloning **a** 10% conditioned media, **b** No conditioned media added, 0.5 cells/well are inoculated in 96-well plates and wells showing colony growth are detected

LDC for both CDM1 and CDM2 required a cloning media that is capable of growing single clones. From a previous work, we already had cloning media developed for CDM1 consisting of serum-free media with components like those in CDM1. In the case of CDM2, we adapted commercial cloning media reported to support good growth for the single clones (Sealover et al. 2013). We attempted to mix the commercial cloning media and CDM2 because we found that commercial cloning media were good for growing single clones but lack of nutrients to maintain cell viability during a long growth period (~ 2 weeks) in 96-well plates. Mixing two media at 25% CDM2: 75% commercial cloning media or at a 1:1 ratio proved enough for growing single clones (Fig. 4). Adding conditioned media to fortify the potential growth factors is known to benefit the growth of single clones in some cases (Sealover et al. 2013; Lim et al. 2013), and adding 10% of conditioned media produced slightly faster growth for single clones, but it did not appear to boost up the clone formation rate significantly. Performing LDC with the best clones from CDM1 and CDM2 platforms resulted in similar clone formation rates (22.7%, 18.5%, respectively) and single clones obtained from LDC were tested in 6-well plates to evaluate the cell-specific productivity. The results of single clone isolation successfully delineated high and low-producing cells (Fig. 3). Overall cell-specific productivity from the CDM1 platform was higher than when using CDM2, and both were superior to CDM3.

### Final clone evaluation

The final clone evaluation was followed by culturing in well-plates directly without any suspension adaptation in shake flask, and microscale bioreactor since the host cells were already pre-adapted to each media as a suspension culture before the transfection.

Top single cell isolated clones obtained from CDM1 and CDM2 were tested (20 clones each) in batch culture, the culture in CDM1 was only able to last up to day 7 before an viability drop while CDM2 was cultured until day 11 before termination. Glucose supplementation was performed, so the reason of viability would not be the glucose depletion, so this data represents the difference between leaner media and richer media. The average of cell-specific productivity among single clones of CDM1 is nearly twofolds higher than CDM2 (13.6 ± 3.7 pg/cell/day and 6.9 ± 2.2 pg/cell/day for CDM1 and CDM2, respectively) (Fig. 3). Inversely, the final productivity was 186.4 ± 31.0 mg/L for CDM1 comparing with 267.4 ± 59.2 mg/L for CDM2, indicating the advantage of developing a production process with richer CDM2.

A fed-batch in microscale bioreactor was performed in the same feed cocktail after confirming this feed (Cell Boost 7a and 7b) worked well in both media. The average of final productivity of 3 batches was 2.3 ± 0.1 g/L and 2.9 ± 0.4 g/L for CDM1 and CDM2, respectively.

## Discussion

Media in CLD have different requirements depending on the process step. Host cell media require the additional components depending on the host cell line, HT is needed for dihydrofolate reductase (*dhfr*), deficient host cell lines (e.g. CHO-DG44, CHO-DUXB11), or glutamine for glutamine synthetase (GS), deficient host cell lines. Furthermore, our experience of adapting host cells in various commercial media found the failure in adapting the cells in some media. CD FortiCHO was powerful media in our recombinant CHO-DG44 cells (Table 2), and was chosen to be tested along CDM1, CDM2, and CDM3, but failed growing in our host cells because of the observation of cell aggregation so had to be halted for this study (data not shown).

Transfection conditions are very different according to the media and convenient to use provided media from the transfection kit to minimize optimization setting the transfection methods. Our set-up polyethylenimine transfection method required OptiPRO SFM to achieve successful transfection, so this media were temporarily used during the transfection step, and changed to the original intended platform media afterwards. As a follow-up study, we tested electroporation to set a transfection method without any media changes and observed that it was possible, but the optimum electroporation conditions (voltage, pulse, and shock no.) differed among CDM1, CDM2, and CDM3 (data not shown).

Selection media are the main media throughout CLD after the transfection is over and adds supplements required for the vector’s selection system. The IgG expression vector in this study simply requires the removal of HT so cells which the vector has been inserted will survive by the presence of *dhfr* gene, and MTX was added to amplify those cells to boost up the cell-specific productivity. Selection reagents such as Geneticin, Puromycin, and Zeocin which the resistance are conferred by *neo* gene, *Pac* gene, and *Sh ble* gene, respectively, are also generally used in CHO cell CLD depending on the vector design. With using CDM1, CDM2 and CDM3 as selection media, a mini-pool screening showed the mini-pool recovery per 96-well plate was nearly 10% with CDM1 while no clones grew with CDM3. Almost all the wells in CDM2 show the growth of mini-pools is too high because an excessive growth ratio per well may lead to a dilution with non-producer cells. This could mask the results of genuine high producer cells, which may be missed (not picked). This can be explained with the principal of the stoichiometry and the material balance on the cell growth. The nutrient is limited in the media, cell growth and production that are determined by a limited nutrient after metabolic process of cells. So, non or low producer cells generally show the high growth rate rather than high producer cells. To identify reasonable growth ratio conditions with CDM2 mini-pool screening, lowering the 96-well plate inoculum cell density or increasing selection pressure in media will be required. In case of CDM3, we assumed that the media were too rich to grow cells in these inoculum cell densities and needed to increase it.

Performance of ClonePix2 screening, as a parallel method for mini-pool screening, showed CDM1/CloneMatrix mixture yielded cell colonies well, and abundant pickable colonies were obtained (11.5% were picked). Similar to a mini-pool screening, CDM2/CloneMatrix mixture yielded too many cell colonies making them too small or too close to neighboring colonies; hence the proportion of pickable colonies was low (40% were picked). The colonies grown in CDM3/CloneMatrix mixture were mostly pickable, but overall number of grown colonies was much fewer than the other conditions. We attempted to optimize the inoculum cell density and CDM2 and CDM3/CloneMatrix ratio conditions after this study. More studying is needed to obtain the clear results but we observed finding an adequate inoculum cell density according to the basal media or its ratio was the most important factor of determining pickable clones. Comparing from the results in this study, CDM2/CloneMatrix mixture required fewer inoculated cells and CDM3/CloneMatrix required more inoculated cells mixture in order to obtain the better results.

The most complicated media in CLD are the cloning media for single cell isolation. Traditional methods simply added serum to the media and can mostly achieve good clonal recovery around 50 ~ 70% in reported studies (Sealover et al. 2013; Zhu et al. 2012; Lim et al. 2013). Achieving good clonal recovery without the presence of serum however is much more complicated, and a report from Sealover et al. (2013) shows that CHO-DG44 is even harder comparing with CHO-S. Mixing conditioned media with the cloning media is a well-used practice and study from Lim et al. (2013) reports, this is because of the secreted growth factors from the CHO cells cultured. However, no clone recovery was obtainable with our recombinant CHO-DG44 with the pre-tested media in Table 2, either with or without adding conditioned media. Mixture of the platform media (CDM1 or CDM2) and commercial cloning media was the best solution in this study, and mixture ratio testing will be required when the individuals will apply new basal media to the CLD platform.

**Fig. 5:**
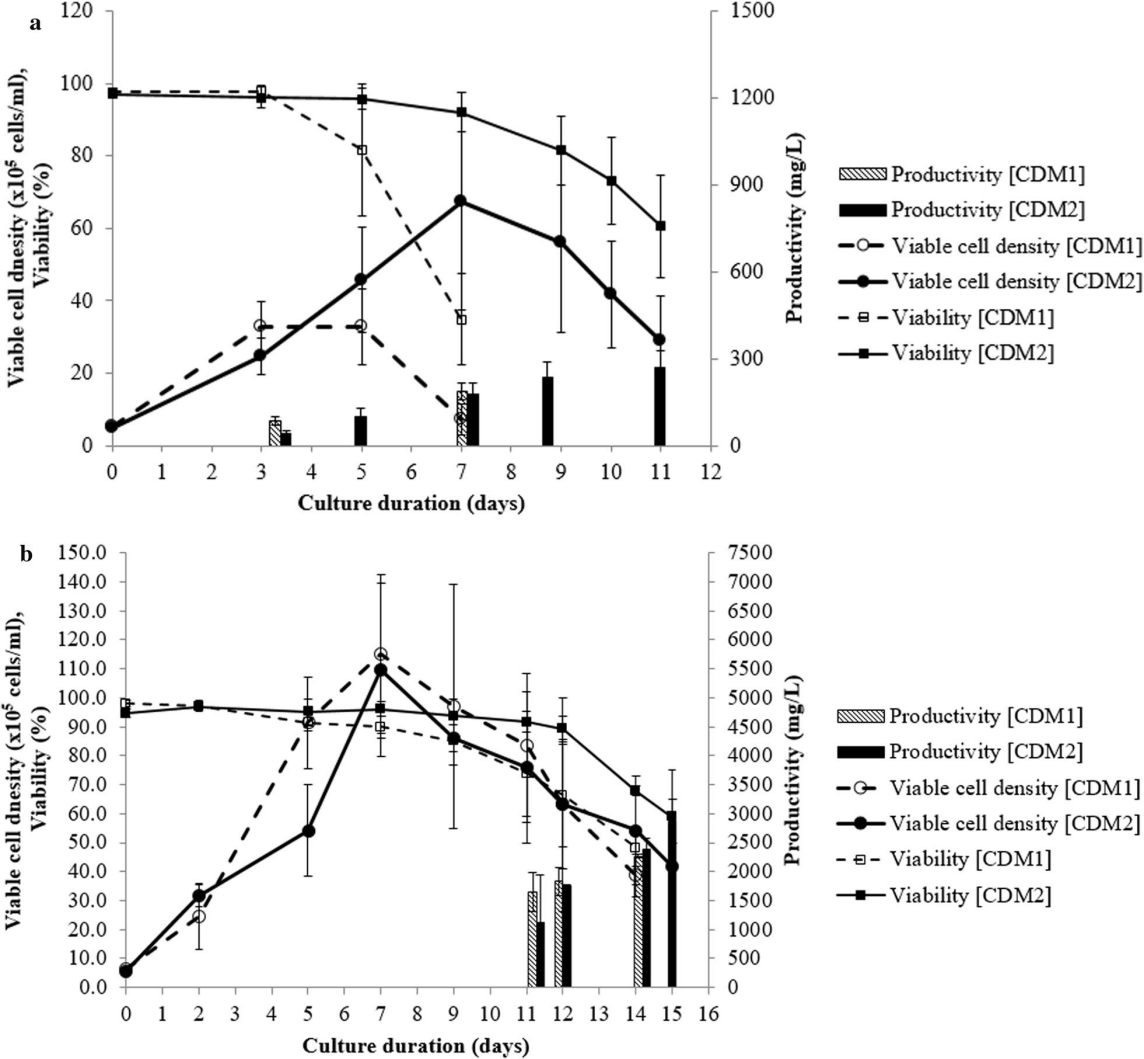
Comparing cell culture data of CDM1 derived clones and CDM2 derived clones **a** batch culture (no feed) data of top clones from CDM1 and CDM2 (average of 20 clones, error bar is standard deviation), **b** fed-batch culture of top clones from CDM1 and CDM2 (average of 3 clones, error bar is standard deviation), CDM1 data used CDM1 as basal media and CDM2 data uses CDM2 as basal media, for fed-batch the same feed cocktail (Cell Boost 7a and 7b) is used in both CDM1 and CDM2 data at the same shots

Final overall evaluation was made with the clones obtained from CLD of CDM1 platform and CDM2 platform. Comparison with a batch and a fed-batch culture was complicated because the different cell lines and media had to be compared. Top clones from CDM1 and CDM2 were tested in a batch culture to make a better comparison, but the cell-specific productivities of CDM1 clones were higher in average (Fig. 3). We found that this was due to difference in screening results were fewer pickable clones in ClonePix2 screening or high growth ratio of mini-pool clones made lower probability of finding high producer clones in CDM2. Clones comparing to a fed-batch (Fig. 5b) were fewer than batch (Fig. 5a) because our fed-batch strategy using Cell Boost 7 generally worked well in both CDM1 and CDM2 in our study, but the variable results were observed at clone–clone comparison regardless of the media. Still general CLD obtains the best working clones with fed-batch conditions, which were the 3 clone comparison in Fig. 5b. and the final productivity in fed-batch for CDM2 was higher than for CDM1 even though the screened clones had overall lower cell-specific productivities. In case of that the production fed-batch culture was performed in CDM1 derived clones with richer media (CDM3 using the same feed cocktail was tested with the clones), the final productivity 2–4 g/L could be observed depending on the clones (data not shown). From the data, we could conclude rich formulated production media such as CDM2 can be successfully used as basal media for CLD showing similar productivity to the CDM1-based cell line.

From the study, we suggest that one of the possible limitations or obstacles using rich media in CLD are the growth limitations in low cell density conditions. Low cell density condition itself is reported to have hindered growth rate (Ozturk and Palsson 1990) due to the decrease in secreted growth factors from the inoculated cells (Lim et al. 2013). But currently, no studies or information about the reason of that richer media can have hindrance to low cell densities. Research such as from Zhu et al. (2012) shows that 1:1 mixture of lean classical media and SFM media achieves better cloning efficiencies, and means that rich media have such effects. Our results also show some limitations from low cell densities in richer media where, the mixture of 1.5× concentrate of CDM2 and CloneMatrix, also the mixture of 1.5× concentrate of CDM3 and CloneMatrix are required and not 2× concentrate (In contrast, the leaner CDM1 worked well with 2× concentrate), mini-pool and ClonePix2 screening needed higher inoculum cell density for CDM3, or a single cell cloning for CDM2 was achieved after mixing with leaner commercial cloning media. Media tend to be optimized to maintain and grow cells better at high cell densities, and are eventually made richer. In the future, research and development of media for the purposes of low cell densities, such as cloning media, will be required.

Herein, we demonstrated that recent rich commercial CHO media formulated for production can be applied as basal media for CLD, serving the cell growth in the initial host cell, screening and selection after transfection, and final production. Developed methods may remove the requirements for cell adaptation to production media after CLD and relieve the clonality issues associated with changing the culture media. This practice will also decrease both time and effort, eliminating the need to adapt cells in new media and optimize the production media process. This will be highly beneficial in modern CLD approaches requiring a shorter development timeline.
